# Amplification Efficiency of Quantitative PCR Reactions is Improved by Addition of Non-Target DNA

**DOI:** 10.1007/s00248-026-02719-0

**Published:** 2026-03-17

**Authors:** Catherine L. Reardon, Daniel K. Manter

**Affiliations:** 1 Soil and Water Conservation Research Unit, United States Department of Agriculture – Agricultural Research Service (USDA-ARS), Adams, OR 97810 USA; 2 Soil Management and Sugarbeet Research Unit, USDA-ARS, Fort Collins, CO 80526 USA

**Keywords:** Quantitative PCR, qPCR, DNA, Amplification efficiency, PCR additive, pUC19

## Abstract

Quantitative PCR (qPCR) is a common, culture-independent method to estimate gene copies at levels as broad as Kingdom and specific as guild, function or species; however, it is dependent upon several factors that affect the amplification efficiency. Amplification efficiency of an individual qPCR reaction is dependent on template type, qPCR product, and assay optimization including primer design. Although qPCR reactions can be optimized, the design of primers to meet guidelines for relatively short amplicon length (75–150 base pairs) is often unfeasible especially when targeting a broad phylogeny of organisms or functional genes. The addition of exogenous non-target plasmid DNA (0.05 ng µL^-1^) as a PCR additive significantly increases the amplification efficiency for long target amplicons up to 723 bp. The method is applicable for different template types including circular and linearized plasmids (24–30% increase) and cell-free DNA extracts (up to 26%), as well as different thermocyclers and qPCR products. As an exogenous DNA source, the pUC19 plasmid provides flexibility with a reduced chance of non-target amplification due to the relatively simple, short, and known DNA sequence. Overall, this method enhances amplification efficiency, reduces template bias, and lowers qPCR variability with a strong benefit for long amplicon assays.

**Supplementary Information:**

The online version contains supplementary material available at 10.1007/s00248-026-02719-0.

## Introduction

Quantification of gene abundance is an important assessment of biological responses across a wide variety of scientific fields (e.g., human, marine, soil) and for use in clinical diagnostics. Within the context of microbial ecology, coupling the community structure and composition with biological function of key species or guilds is an important goal. Quantitative PCR is a rapid, cost-effective, simple, and commonly used method to estimate gene abundance of targets as broad as Kingdom and as specific as guild, physiological function or species. In some [1, 2], but not all environments [3], assessment of specific functional gene sequences can provide a powerful estimate of potential activity. For example, qPCR estimates of the gene abundance for nutrient cycling [1, 4] and other soil enzymes [2] can be used as a proxy for soil function due to the strong correlations between gene copy number and enzyme activity.

The qPCR method has broad applicability to numerous gene targets and templates based on primer design; however, meeting requirements for primer specificity, phylogenetic breadth (when required) and assay performance (e.g., amplification efficiency) may not always be possible. Strict guidelines for primer design typically suggest an amplicon length less than 200 bp to maximize amplification efficiency [5–7]; however, this limits flexibility for qPCR assays to use well-established primer sets that target longer amplicons and coupling of gene abundance measurements with community sequencing results, especially when capturing a wide range of taxonomic diversity. Although target specificity is an immediate concern with qPCR assay development, the amplification efficiency is integral to reliable estimates [8].

Amplification efficiency can refer to different aspects or calculations in qPCR analyses. Most commonly, efficiency refers to the slope of a standard curve in which quantification cycle (C_*q*_) or cycle threshold (C_*t*_) are plotted against the log10 values of the concentrations or dilution factors [9]. This method is sensitive to variability in the standard curve and errors in dilution steps. Comparatively, amplification efficiency can refer to the log-linear phase of the amplification curve of a single reaction [9] independent of errors in the dilution series. For this study, amplification efficiency refers to the efficiency of a single reaction rather than the slope-based calculation.

Good amplification efficiency is a product of optimal conditions including target sequence [10]; primer design for amplicon length, conformation [9] and stability [11, 12]; and other factors of instrumentation [10], thermocycling conditions, sample matrix, reagents, and concentrations [13]. In a perfect reaction, the amount of target copies will double with each amplification reaction for an efficiency value of 2 (or 100%); however, despite optimization efforts, differences in amplification efficiency can occur due to the type of DNA extract (i.e., cell-free extracts, PCR products, plasmids) making accurate estimations unfeasible [10, 11, 14, 15]. This can be exacerbated when the type of DNA used for the unknown samples (i.e., mixed-template, cell-free extracts) differs from that of the standard curve (e.g., plasmids, PCR amplicons or genomic DNA) or with low concentration templates that are more susceptible to errors from dilution, PCR inhibitors, and sample loss [16–20]. Maintaining uniform amplification efficiency across samples and standards is critical and even more difficult when samples and standards come from different matrices or sources (e.g., soils, cultures, tissue).

Despite methodological challenges, qPCR remains an indispensable tool bridging research and diagnostics [21, 22]. Although new tools for quantification are becoming more adopted, advances in existing techniques remain necessary as qPCR provides the benefits of high-throughput capacity, versatility, and relatively low cost. While qPCR has played a central role in microbial ecology, modern application in quantitative community profiling brings new significance to this foundational technique. For example, quantitative analyses can be applied to soil microbial community sequence results to combat interdependency of relative sequence abundances [23, 24] by normalization to absolute abundance [25–27]. This methodology, however, has not yet been widely adopted due in part to primer limitations and effects of template source on amplification efficiency, both of which commonly plague qPCR assays [10, 27]. As sequencing technologies increase the capacity for long template reads, the constraints of amplicon size for qPCR remain. Therefore, improving the amplification efficiency of qPCR for longer amplicons and different DNA sources is a critical step in the development of novel applications in microbial ecology to assess complex communities.

This work demonstrates that amplification efficiency is strongly affected by amplicon length and template concentration, and that the simple addition of non-target DNA can improve efficiency, increase the amplicon target length amenable to qPCR, and reduce the technical and between-run variability. The results are specific to microbial- and soil-derived DNA templates but may be applicable to other DNA sources. Improvement of amplification efficiency for long amplicons expands the ability to generate robust qPCR assays with particular relevance to quantitative DNA sequencing and functional gene analysis.

## Materials and Methods

### DNA Template

Quantitative PCR reactions were performed using circular plasmid, linearized plasmid, and genomic DNA. Plasmid templates were generated by cloning PCR amplicons of various lengths into the pCR4 plasmid using the TopoTA Cloning Kit for Sequencing (Invitrogen, Carlsbad, CA). The PCR amplicons were from functional nitrogen cycling genes or 16S genes amplified from soil or genomic DNA (Supplemental Table 1). Plasmid extracts were quantified using the Quanti-iT dsDNA Assay (HS) with the Qubit Fluorometer (Invitrogen) and verified for the correct insert size by restriction digest with EcoRI-HF (New England Biolab, Ipswich, MA) and analysis on the Agilent Bioanalyzer 2100 (Agilent Technologies, Santa Clara, CA). Linearized plasmids were produced by restriction digest with SspI-HF (New England Biolab), purified with AMPure XP beads (Beckman Coulter, Indianapolis, IN), and verified for single cut sites using the Bioanalyzer. Plasmids were diluted in molecular grade water to copies per µL based on plasmid size and concentration.

Genomic templates were prepared from soil and the bacterial ammonia oxidizer *Nitrosomonas europaea* ATCC 19718. Soil-derived DNA was extracted from agricultural fields in Pullman and Chelan, WA, USA using the PowerLyzer PowerSoil DNA Isolation Kit (Qiagen, Germantown, MD) or Ultraclean Soil DNA Isolation Kit (Mo Bio Laboratories, Carlsbad, CA), respectively. Soil DNA was diluted 1:20 in molecular-grade water. The *N. europaea* genome encodes two copies of the *amoA* gene [28]. The genomic DNA (gDNA) was diluted to 1.2 × 10^5^ and 1.2 × 10^3^ genomes µL^− 1^ to produce templates of 2.4 × 10^5^ and 2.4 × 10^3^ *amoA* gene copies µL^− 1^ prior to analysis. DNA templates were stored and diluted in low retention LoBind tubes (Eppendorf, Enfield, CT) to reduce DNA loss through adhesion [16].

### Quantitative PCR

#### Functional Gene Abundance

To evaluate differences in amplification efficiency between DNA types, the bacterial ammonia monooxygenase (*amoA*) gene was amplified from genomic DNA (*N. europaea*), soil DNA, and circular plasmid for the standard curve. Diluted soil DNA was confirmed to be free from effects of PCR inhibitors per Reardon, et al. [29]. Quantification was performed with the bacterial amoA-1F and amoA-2R primers [30] that target a region of approximately 491 bp. The standards were prepared from a 10-fold dilution series of circular plasmid containing a cloned *amoA* gene fragment (Supplemental Table 1). The qPCR reactions were 10 µL volumes containing 1X PowerSYBR Green PCR Master Mix (Life Technologies, Carlsbad, CA), 0.2 µM forward and reverse primers, 1 µL DNA template or water as a no template control, and either 0 (additive-free) or 0.05 ng µL^− 1^ (final concentration) pUC19 plasmid additive. The pUC19 plasmid was extracted in house or sourced commercially (New England Biolabs). All qPCR template concentrations are provided in copies or ng µL^− 1^ qPCR reaction. Standards ranged from 1.0 × 10^5^ to 1.0 × 10^1^ copies µL^− 1^ reaction and *N. europaea amoA* gene copies were 2.4 × 10^4^ (high) and 2.4 × 10^2^ (low) copies µL^− 1^ reaction. All reactions including the no template control were performed in triplicate on a 96-well plate and the experiment repeated up to seven times. Thermocycling was performed on the StepOnePlus Real Time PCR System (Applied Biosystems, Carlsbad, CA) in a 3-step reaction with 95 °C for 10 min, 40 cycles of 95 °C for 15 s, 60 °C for 1 min, and 76 °C for 30 s prior to the read. Assay conditions were optimized empirically in additive-free master mix for primer concentration, annealing temperature, and the extended elongation step due to the amplicon size (> 400 bp). The standard curve was generated in StepOnePlus software by linear regression of the cycle threshold (C_*t*_), or cycle number at which fluorescence exceeds a predefined threshold (*t*), with the starting template concentrations of the standards. Gene copy number was calculated in StepOnePlus from the standard curve with different threshold settings (0.1, 0.5, 1*)* as:

1 $$\text{Copy number} = 10^{\wedge}((C_t - \mathrm{y-intercept}) / \mathrm{slope})$$

#### Amplicon Length Effects

The effect of amplicon size on amplification efficiency was evaluated using circular plasmid template with a range of insert sizes. Reaction conditions were empirically optimized for PowerSYBR Master Mix including primer concentration, volume and thermocycling protocol. Amplification was performed using primers pCR4-265F (5’—AGT CCT GCA GGT TTA AAC GAA—3’) and pCR4-312R (5′—ATA GGG CGA ATT GAA TTT AGC G—3’) developed specific to the pCR4-TOPO plasmid within 15 bp of the cloning site (Supplemental Table 1). The amplicons ranged in size from 239 to 723 bp and in GC content from 51 to 62%. The qPCR reactions were prepared in 10 µL volumes with 1X PowerSYBR Master Mix, 0.4 µM pCR4-specific primers, 1 µL plasmid template or water as a no template control, and 0 (additive-free) to 2 ng µL^− 1^ pUC19 additive (final concentration). Plasmid templates were 1 × 10^3^ copies per µL^− 1^ reaction. Thermocycling was performed using the StepOnePlus thermocycler in a two-step reaction with 95 °C for 10 min denaturation, and 40 cycles of 95 °C for 15 s and 58 °C for 1 min. All reactions including the no template control were performed in triplicate and the assays conducted up to 9 times across several years. The experiment was repeated with different thermocycling platforms (QuantStudio Pro 6 [Applied Biosystems] and LightCycler 96 [Roche, Indianapolis, IN]), an alternative additive (salmon sperm DNA; Invitrogen), and different qPCR master mix products of varying composition. The master mix products were Maxima SYBR Green/ROX qPCR Master Mix with Maxima Hot Start Taq DNA Polymerase and dUTP (Thermo Scientific, Waltham, MA); PowerUp SYBR Green Master Mix for qPCR with Dual-Lock Taq DNA Polymerase, dUTP, and uracil-DNA glycosylase (UDG) (Applied Biosystems); and QuantiNova SYBR Green PCR Kit with QuantiNova DNA Polymerase (no dUTP) (Qiagen) in addition to PowerSYBR Master Mix with AmpliTaq Gold LD polymerase and dUTP. Amplicons were assessed by melt curve analysis or gel electrophoresis with 1.8% agarose, 5 µL qPCR reaction, and 6 µL 100 bp ladder (New England Biolabs).

### LinRegPCR

Amplification efficiencies of the individual reactions were calculated from the slope of the amplification curve using LinRegPCR v2021.2 [31, 32]. The amplification data (Rn), which is the reporter dye (SYBR) signal normalized to the passive reference dye (ROX), was used for analysis per the LinRegPCR user manual. The Rn data was imported into LinRegPCR using the StepOnePlus and ViiA7 (ABI) data file format with selection of DNA-binding dye chemistry, ds-DNA amplification, and no baseline correction. Data were analyzed using fit options (individual) of 4–6 points with best correlation coefficient, common threshold, individual Window-of-Linearity, and manual baseline correction. Baselines were generally left as calculated in the software except in rare cases where one of the three well replicates was a clear outlier and efficiency was improved using manual baseline correction. Means and standard deviations of triplicate reactions were calculated in Microsoft Excel. Theoretically, a perfect amplification efficiency would be 2 in which DNA copies double with each cycle. An efficiency of 1 indicates a failed reaction. The percent amplification efficiency was calculated as:

2 $$\begin{array}{l} \:\%\:Amplification\:efficiency\\=\:(individual\:amplification\:efficiency-1)\times\:100 \end{array}$$

### Statistical Analysis

Differences in the amplification efficiency for qPCR reactions targeting the bacterial *amoA* functional gene was assessed by averaging three well replicates in a 96-well assay format for 5 to 7 different experimental runs. Data were analyzed via the generalized linear mixed model (Proc GLIMMIX) in SAS version 9.4 (SAS Institute Inc., Cary, NC) with experimental run as the random variable. Means separation was performed with Tukey adjustment. Gene quantification was performed with the StepOne Software (v2.3; Applied Biosystems) for a single plate run with different threshold settings. Statistical analysis of the effects of pUC19 additive and threshold settings on gene quantification were analyzed with Proc GLIMMIX with reaction (i.e., individual wells) as the random variable and Tukey adjustment for means separation. The relationships between amplification efficiency and amplicon size were evaluated using plasmid templates containing inserts of varying lengths, based on replicate means from 11 experimental runs, with experimental run as the random variable. Linear regressions were conducted across all treatments and parameters in Microsoft Excel. In all analyses *P* < 0.05 was considered significant.

## Results and Discussion

Forging discoveries with molecular analyses necessitates development of new methods but also the improvement of established and validated techniques, such as qPCR. In ecological studies, the requirements for analysis of communities at broad phylogenetic, guild-based, or functional gene level add layers of difficulty in gene quantification due to challenges in primer design including the inability to match primers for DNA sequencing or to achieve adequate amplification efficiency. General guidelines for qPCR primer design are restrictive, especially when designing primers to capture phylogenetic diversity. For example, primers should be between 18 and 24 bp, target relatively short amplicon lengths, have minimal guanine repeats with GC contents between 50 and 60%, and melt temperatures between 55 and 65 °C with no more than 2 °C difference [12, 33]. Although *in silico* tools have greatly facilitated primer design [34–37], mixed target samples such as soil raise unique problems due to the complexity of target genes and availability of homologous regions [34, 38, 39]. Degeneracy can broaden the detection of diverse templates, but it is not recommended for qPCR due to amplification bias [33, 40]; however, this requirement may limit target inclusivity especially when used for community analyses.

Experimental and mathematical methods have been developed to correct quantification errors due to differences in amplification efficiency or to push limits of detection. One option has been to add chemical or nucleic solutions to the master mix [18] or as diluents for the standard curve [16, 41]. Although some additives positively affect the efficiency of the standard curve for qPCR, the effects on amplification efficiency are often not reported [21] and the target regions short (66–77 bp) [41] or unspecified. Mathematical methods [9, 14, 19] can be used for *post hoc* correction of differences in amplification efficiency. For this study, LinRegPCR was used to calculate the amplification efficiency for individual reactions to assess the effects of amplicon size, DNA template type and concentration, and the potential for *in tube* correction using a plasmid additive. Amplification curves with efficiencies of 70–110% (1.7–2.1 efficiency) were considered acceptable based on the assessment and positive control samples of Ruijter, et al. [9].

### Sample Type and Concentration Affect Amplification Efficiency

**Fig. 1:**
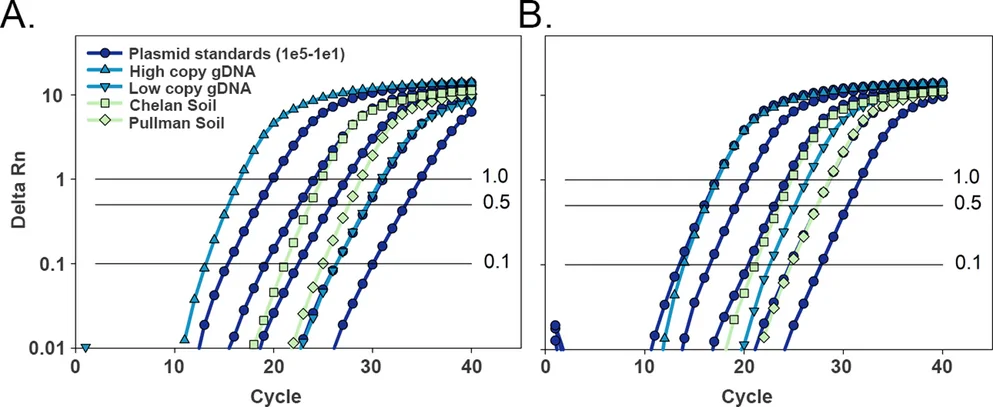
Amplification curves of qPCR reactions performed with PowerSYBR Master Mix on the StepOnePlus platform targeting bacterial ammonia monooxygenase gene (*amoA*; 491 bp) from templates of plasmid (circular pCR4 plasmid in 10-fold dilutions from 1 × 10^5^ to 1 × 10^1^ copies *amoA* µL^−1^ qPCR reaction), *N. europaea* gDNA (high copy, 2.4 × 10^4^ copies *amoA* µL^−1^ reaction; low copy, 2.4 × 10^2^ *amoA* copies µL^−1^ reaction), and soil DNA from Chelan, WA and Pullman, WA. Reaction master mix was either (A) without additive (0 ng µL^−1^) or (B) with pUC19 additive (0.05 ng µL^−1^). Data were plotted as the average of three technical replicates. Horizontal lines indicate threshold values of 1, 0.5 and 0.1 delta Rn (reporter signal). Gene copy estimates are provided in Table 2

Quantitative PCR commonly uses genomic DNA or manufactured DNA (i.e., amplicons, plasmid inserts, or synthesized oligonucleotides) as a standard curve template. The use of manufactured DNA can be desirable in studies where genomic template is unavailable or unfeasible such as for rare or difficult to culture microorganisms; however, template type can have a significant effect on amplification efficiency [11, 14]. Early PCR primers designed for the bacterial *amoA* gene produce an amplicon of approximately 491 bp in length. When quantifying the functional gene, template type (i.e., plasmid, soil DNA, genomic DNA) had a visible effect on the slope of the amplification curve indicating that the amplification efficiency differed by template type and concentration (Fig. 1A). Noticeably, soil DNA and the high copy gDNA (2.4 × 10^4^ *amoA* copies µL^− 1^ reaction) had low but acceptable amplification efficiencies (71–79%) whereas the low copy gDNA (2.4 × 10^2^ *amoA* copies µL^− 1^ reaction) and circular pCR4 plasmid had poor efficiencies ranging from 48 to 54% (Table 1).

**Table 1:** Amplification efficiency of qPCR reactions for the bacterial ammonia monooxygenase gene (*amoA*) from circular pCR4 plasmid, *N. europaea* gDNA, or soil DNA templates. Reactions were performed on the StepOnePlus platform with a single primer set and PowerSYBR master mix without (0 ng µL^-1^) or with pUC19 additive (0.05 ng µL^-1^). Amplification efficiencies were calculated using LinRegPCR. Values indicate mean amplification efficiency (%) and standard deviations (parentheses) of triplicate qPCR reactions across the specified number of qPCR runs (i.e., experimental replicates). Shared letters for each column indicate the values are not significantly different (*P* > 0.05).

Template type	Copy no. or source^†^	DNA conc. (ng µL^− 1^ reaction)^‡^	qPCR runs	% Efficiency		Difference^§^
				No additive	pUC19 additive	
pCR4 plasmid	1 × 10^1^	4.9 × 10^− 8^	6	53.4 (6.9) B	76.9 (4.3) A	23.5***
	1 × 10^3^	4.9 × 10^− 6^	7	48.3 (4.8) B	78.7 (8.1) A	30.3***
	1 × 10^5^	4.9 × 10^− 4^	7	48.6 (5.3) B	78.2 (7.4) A	29.6***
*N. europaea* gDNA	Low	3.7 × 10^− 4^	6	53.5 (4.7) B	79.4 (3.4) A	26***
	High	3.7 × 10^− 2^	7	78.8 (4.9) A	82.9 (5.6) A	4.1
Soil gDNA	Chelan	3.6 × 10^− 2^	5	71.4 (14.6) A	74.6 (11.5) A	3.3
	Pullman	7.6 × 10^− 2^	5	76.4 (3.8) A	82.0 (7.2) A	5.6

^†^Gene copy number µL^− 1^ in the qPCR reaction for plasmid and genomic templates, or location of soil collection in Washington, USA. Low copy *N. europaea* gDNA was 2.4 × 10^2^ and high copy gDNA was 2.4 × 10^4^ copies *amoA* µL^− 1^ reaction

^‡^Final DNA concentration in the qPCR reaction

^§^Difference in percent amplification efficiency (pUC19 additive – no additive). Significance is shown as *** *P* < 0.0001, ** *P* < 0.001, *P < 0.01

Amplification efficiencies were calculated using LinRegPCR. Values indicate mean amplification efficiency (%) and standard deviations (parentheses) of triplicate qPCR reactions across the specified number of qPCR runs (i.e., experimental replicates). Shared letters for each column indicate the values are not significantly different (*P* > 0.05)

In addition to differences in the types of DNA, the concentration of DNA in the reaction was also variable and reflective of the trend in amplification efficiency. DNA concentrations in the reactions ranged several orders of magnitude from the lowest samples (4.9 × 10^− 8^ ng µL^− 1^) which included pCR4 plasmid templates and low copy gDNA, to the high concentration soils (Chelan, 3.6 × 10^− 2^ ng µL^− 1^; Pullman, 7.6 × 10^− 2^ ng µL^− 1^) and high copy gDNA (3.7 × 10^− 2^ ng µL^− 1^) (Table 1). Of these, only the DNA concentration at or above 3.6 × 10^-2^ ng µL showed no reduction in PCR efficiency when pUC19 was excluded from the reaction mix. Based on the observation that amplification efficiency was sensitive to DNA concentration rather than type, we hypothesized that low efficiency could be ameliorated by adding exogenous DNA to the master mix rather than correction with *post-hoc* analyses.

### Exogenous DNA Enhances Amplification Efficiency

The pUC19 plasmid was selected as an exogenous DNA additive due to its small size (2868 bp) and known DNA sequence thus minimizing the potential for primer-complementarity and non-target amplification. Early tests with different concentrations of *in-house* extracted pUC19 identified 0.05 ng µL^− 1^ as a starting point for optimization. Unlike studies that used carrier DNA for the dilution of standards [16], plasmid was added to the entire master mix used for both standards and unknown samples.

Master mix with the pUC19 additive produced similar amplification curves and amplification efficiencies (> 75%) for the *amoA* gene across all template types and concentrations (Fig. 1B, Table 2). The amplification efficiencies of low concentration samples (i.e., pCR4 plasmids and low copy gDNA) increased significantly by 24–30% in reactions with the pUC19 additive as compared to those without. Positive but non-significant effects (3.3–5.6%) of pUC19 additive were observed for the efficiencies of high concentration samples (soils and high copy gDNA) (Table 1). Melt curve analysis indicated that the positive effects of pUC19 additive on amplification efficiency were not due to non-specific amplification as the no template controls remained negative and the melt curves were similar for each template regardless of the additive (Supplemental Fig. 1).

**Table 2:** Estimated abundance of bacterial ammonia monooxygenase gene (*amoA*) from two soil DNA extracts and *Nitrosomonas europaea* gDNA as quantified on the StepOnePlus platform with a single primer set in qPCR reactions using PowerSYBR master mix without additive (0 ng µL^−1^) or with pUC19 additive (0.05 ng µL^−1^). Copy number was calculated using StepOne software (v2.3) with different fluorescence threshold setting values for a single qPCR run. Shared letters for each column indicate the values are not significantly different (*P* > 0.05). Amplification curves are shown in Fig. 1

Treatment	Fluorescence threshold setting	pCR4 standard curve efficiency (%)	*amoA* copy number per µL reaction†				Fold-change^‡^	
			Soil DNA		*N. europaea* gDNA		*N*. *europaea amoA*	
			Chelan	Pullman	High	Low	High	Low
No additive	0.1	83.95	2.68 × 10^3^ B	2.52 × 10^2^ C	2.88 × 10^5^ C	1.10 × 10^2^ C	12.01	0.46
	0.5	84.31	4.23 × 10^3^ A	3.82 × 10^2^ B	5.37 × 10^5^ B	1.25 × 10^2^ C	22.38	0.52
	1	84.52	5.23 × 10^3^ A	4.62 × 10^2^ A	6.87 × 10^5^ A	1.31 × 10^2^ C	28.63	0.55
	%CV		32	29	40	9		
Additive	0.1	87.66	7.37 × 10^2^ C	7.36 × 10^1^ D	6.64 × 10^4^ D	2.85 × 10^2^ B	2.77	1.19
	0.5	86.03	8.13 × 10^2^ C	7.63 × 10^1^ D	8.07 × 10^4^ D	3.10 × 10^2^ AB	3.36	1.29
	1	85.80	8.30 × 10^2^ C	7.78 × 10^1^ D	8.60 × 10^4^ D	3.17 × 10^2^ A	3.58	1.32
	%CV		6	3	13	6		

^†^Measured *amoA* copies µL^− 1^ of the qPCR reaction. Location of soil collection in WA, USA is shown. *N. europaea* gDNA was 2.4 × 10^4^ (high) and 2.4 × 10^2^ (low) copies *amoA* µL^− 1^ qPCR reaction. Calculations were based on a standard curve of circular plasmid which is reported to overestimate copy number [42]. %CV, percent coefficient of variability for copy number data

^**‡**^Fold-change was calculated as measured copy number divided by the expected copy number

### Exogenous DNA Reduces Quantification Bias

It is well recognized that differences in amplification efficiency between standards and samples can have a large effect on quantification [8, 10, 16]. The pUC19 additive affected both the measured abundance of the *amoA* gene and the sensitivity of the estimates to the fluorescence threshold setting used for the analysis (Table 2). The threshold value significantly affected the copy number estimates of *amoA* with greater variability in reactions without pUC19 additive (28% average CV) as compared to those that received the additive (7% average CV) (Table 2). Estimates with the greatest sensitivity to the threshold setting were those in which the amplification efficiency varied significantly from the pCR4 plasmid standard (i.e., high concentration templates without pUC19 additive) (Tables 1 and 2) as demonstrated by others [8]. With the pUC19 additive, estimates of *amoA* copy number were similar regardless of threshold setting for the high copy templates suggesting a reduced sensitivity to the threshold. Comparatively, significant differences in estimates were observed between 0.1 and 1 threshold settings for the low copy gDNA template indicating that even though pUC19 can improve amplification efficiency, care must still be taken for analyses of varying template type or concentration.

The *N. europaea* gDNA template was used to contrast template concentrations and differing amplification efficiencies on gene estimates. Quantification using circular plasmid standards, as employed here, has been reported to overestimate gene abundance up to 8-fold [42]. The overestimation is suggested to occur from suppression of PCR by the supercoiled plasmid structure within the first several qPCR cycles [42]. Overestimation was observed for the high copy gDNA template in additive-free reactions in which gene copy estimates were 12- to 29-fold greater than the input template (Table 2). The pUC19 additive greatly decreased the overestimation in which estimates of the high copy gDNA differed from the input template by 2.8- to 3.6-fold. Gene estimates for the low copy gDNA template which had similar amplification efficiencies to the standards, provided reliable gene estimates regardless of pUC19 additive with less than 0.6-fold difference from the input concentration (Table 2). Sample-to-sample variation in template concentration had significant effects on quantitative outcomes, thus highlighting the importance of amending the entire master mix with pUC19 additive rather than using it as a standard curve diluent [16].

### Exogenous DNA Enhances Amplification Efficiency for Fragments of Varying Length

**Fig. 2:**
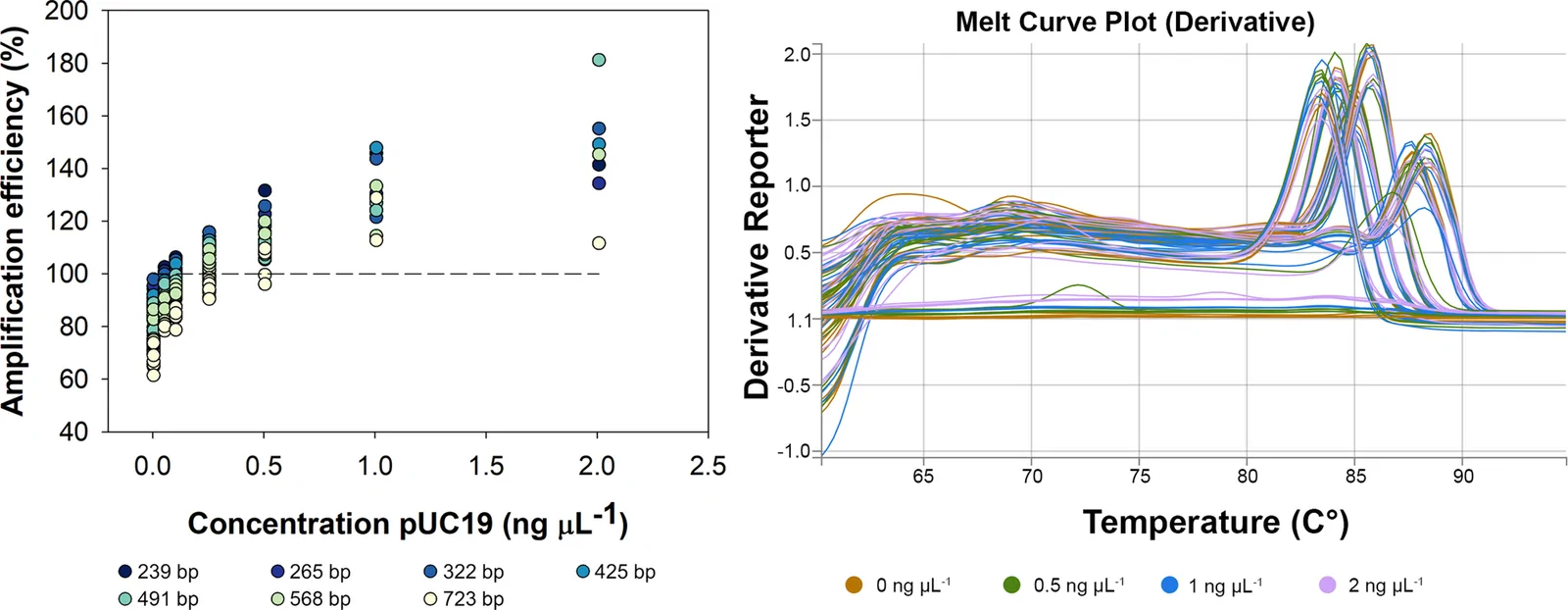
Effect of pUC19 on the amplification efficiency of circular plasmid template with varying insert sizes. A) Amplification efficiencies in qPCR reactions performed on the StepOnePlus platform with PowerSYBR Master Mix and different concentrations of the pUC19 plasmid. Data points are the average of three technical replicate (wells) for up to 13 different qPCR runs conducted over three years (0-0.025 ng μL^-1^, n>9; 0.5-2 ng μL^-1^, n=1-4). Amplification efficiency of qPCR reactions was calculated from the amplification data using LinRegPCR. B) Melt curve analysis of amplicons ranging in size (239-723 bp) in qPCR reactions containing 0, 0.5, 1, and 2 ng μL^-1^ pUC19 additive. Amplicon size is indicated by peaks and flat lines at bottom are the no template controls.

The effects of exogenous, non-target DNA on amplification efficiency were tested on reactions with varying target lengths by amending the qPCR reactions with a range of concentrations of the pUC19 additive. To mitigate primer effects, the different amplicon sizes were generated using a pCR4 plasmid with varying insert sizes so that the same primer could be used for all qPCR reactions thus minimizing primer bias. In the additive-free reactions, a negative effect of amplicon size on amplification efficiency was clearly observed in which the shortest amplicons (239 and 265 bp) were on average 88–90% efficient (*n* = 13 runs) compared to longer amplicons (> 372 bp) ranging from 70 to 80% efficient (*n* = 12–13 runs) (Fig. 2A). The pUC19 additive significantly increased amplification efficiency across all amplicon lengths (*P* < 0.001) although pairwise comparisons between additive-free (0 ng µL^− 1^) and the lowest additive concentration (0.05 ng µL^− 1^) were not significant for the shortest fragments (< 372 bp) that already had high efficiency (> 88%). The increased efficiency was not due to non-specific amplification as all reactions for varying length produced amplicons of the appropriate size regardless of the additive (0 vs. 2 ng µL) and no template controls remained negative for amplification (Fig. 2B, Supplemental Fig. 2). The addition of pUC19 additive greater than 0.1 ng µL^− 1^ increased efficiency above 100% for most amplicon sizes which indicates an error in the baseline estimate being set too high [32]. It is likely the high pUC19 concentrations increased the baseline fluorescence due to non-target (amplicon) association, among other factors [32]. Manual baseline correction is an option in LinRegPCR to “rescue data”; however, the baselines were generally set using strictly continuous log-linear phase option to simply assess the amplification efficiencies although some runs produced data requiring a relaxed baseline that allowed jumps and drooping points.

**Fig. 3:**
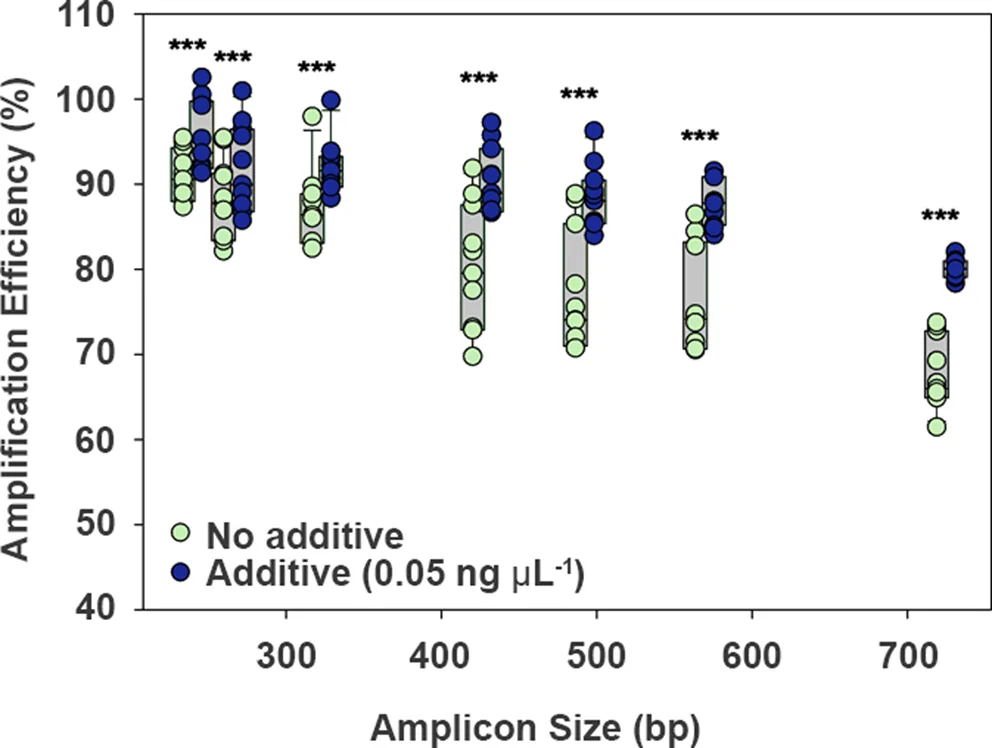
Effect of pUC19 additive on the amplification efficiency of circular plasmid templates with varying insert sizes. Amplification was performed using a StepOnePlus thermocycler with PowerSYBR Master Mix and either no additive (0 ng µL^− 1^) or pUC19 additive (0.05 ng µL^− 1^). All no template control reactions were negative (data not shown). Data points are the average of three well replicates for 11 qPCR runs (*n* = 11) conducted over three years (2021, 2023, 2025). Pairwise comparisons for each amplicon size were significant at *P* < 0.001 shown as ***

The concentration of pUC19 additive of 0.05 ng µL^− 1^ was selected for further optimization because it increased efficiency from the additive-free reactions with minimal reactions exceeding 100%. In pairwise comparisons by amplicon length, the qPCR additive (0.05 ng µL^−^^1^) significantly increased amplification efficiency for all base pair lengths (Fig. 3). The efficiency of the 723 bp, which far exceeds the recommendations for qPCR, showed an increase of over 10% by the addition of the pUC19 additive (average of 68% for additive-free to 80% for additive-amended). Notably, the pUC19 additive reduced the run-to-run variability in amplification efficiency for longer amplicon lengths (320 bp and greater) as demonstrated by 30–70% lower standard deviation between runs.

**Fig. 4:**
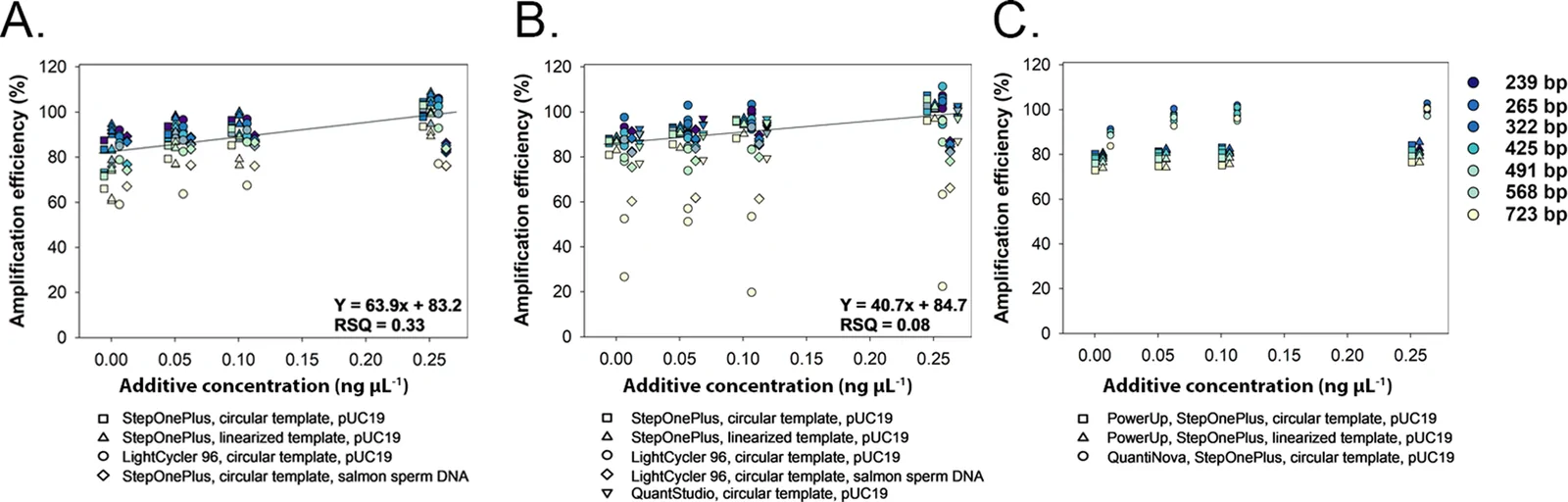
Effect of exogenous DNA additives on the amplification efficiency of plasmid template with varying insert sizes using different products, thermocyclers and plasmid forms. Reactions were prepared with (**A**) PowerSYBR, (**B**) Maxima, or (**C**) PowerUp and QuantiNova Master Mix either without or with additives of circular pUC19 plasmid, linearized pUC19 plasmid, or salmon sperm DNA at 0 to 0.25 ng µL^− 1^ concentration. No template control reactions were negative (data not shown). Data points are the average of three well replicates for 1–2 qPCR runs (*n* = 1 or 2). Regressions across all treatments are shown for PowerSYBR and Maxima Master Mix products

To evaluate whether the method has broader applicability, the qPCR reactions were performed with additional master mix products, platforms, template types, and additives. The focus on testing these different parameters was to contrast the effect of the additive rather than to achieve maximum efficiency, which would require further optimization for each parameter. Similar to previous analyses, varying template size was achieved using plasmids with different cloned inserts producing amplicons of 239–723 bp. The PowerSYBR Master Mix showed general trends of enhanced amplification efficiency (slope = 63.9) with the additive regardless of instrument platform (StepOnePlus vs. LightCycler96), template type (circular or linearized pCR4 plasmid) or additive type (pUC19 or salmon sperm DNA) (Fig. 4A). In all treatments, the amplicon size had a downward distribution in amplification efficiency across all additive concentrations. Similarly, amplification efficiency with Maxima Master Mix was also enhanced by the additive but to a lesser degree (slope = 40.7) than PowerSYBR Master Mix (Fig. 4B). Unlike PowerSYBR, the downward trend in efficiency by amplicon size with Maxima was less apparent in the lower base pair lengths suggesting this master mix may be less sensitive to size. The pUC19 additive showed promise for QuantiNova SYBR Green PCR with an upward trend in efficiency between the additive-free and additive-amended (0.05 ng µL^− 1^) assays (single run) but no effect for PowerUp SYBR Green Master Mix (Fig. 4C) suggesting that product-based differences (e.g., proprietary chemistries, polymerase, dUTP/UDG) affect the additive performance. The three platforms also provide an interesting contrast for performance since the Applied Biosystems platforms (StepOnePlus and QuantStudio) use the internal reference dye, ROX, whereas the LightCycler 96 does not. The use of ROX affects the data input values for LinRegPCR. Reference-normalized data with technical background correction is used for platforms such as Applied Biosystems whereas the Roche LightCycler 96 uses data corrected only for technical background. For both types of systems, the pUC19 additive was successful in enhancing efficiency.

### Biophysical and Biochemical Effects of Exogenous DNA on PCR

The positive effects of pUC19 DNA additive on amplification efficiency likely stem from a combination of biophysical and biochemical mechanisms that collectively influence molecular interactions [43–45] and preserve polymerase activity [46]. As a high-molecular-weight polyanion, additive DNA may act as a macromolecular crowder by increasing the excluded volume and effective concentrations of PCR components [44]. In addition, molecular crowders, such as PEG, can enhance PCR efficiency by increasing activity of polymerase [44], the binding of enzyme with DNA [43, 47], the rate of primer annealing, and DNA duplex stability [45]. Additionally, non-target DNA may provide a competitive sink for trace inhibitors or tube-derived contaminants that would otherwise sequester polymerase and decrease the fraction of reactants available for productive extension [46]. Polypropylene plastics have long been observed to absorb DNA and induce conformational changes and degradation [20, 48, 49]. Although LoBind (or low adhesion) tubes were used for template preparation to reduce DNA loss [16], DNA adhesion is still expected to occur with more pronounced effects on recovery of low concentration samples [16, 20]. Addition of non-target DNA may saturate the hydrophobic binding sites on polypropylene surfaces, preventing adsorption of both polymerase and low-copy template molecules [20] thus increasing the effective concentration of reactants throughout cycling [45]. Together, these effects shift the reaction environment toward less template loss, more efficient primer binding, and improved polymerase performance, thus improving amplification efficiency particularly with low DNA concentration samples (in this study, < 3.6 × 10^− 2^ ng µL^− 1^ per reaction).

## Summary

The addition of non-target DNA to SYBR-based qPCR assays is another tool in the optimization toolkit especially for challenging templates; however, empirical qPCR optimization (e.g., extension time, primer concentrations, annealing temperature, salts) remains an essential step. In this study, the simple addition of pUC19 plasmid at 0.05 ng µL^− 1^ reaction to the qPCR master mix reduced variability and increased the amplification efficiency of both long amplicons and low concentration samples for different qPCR platforms and master mixes. Further optimization, including additive concentration, may be necessary for other SYBR-based qPCR products, assay parameters, and DNA sources as this study relied on microbial- and soil-derived templates. The addition of non-target DNA provides an opportunity to expand the application of qPCR to both new and well-established (e.g., 16S rRNA) assays that are outside the previously recommended amplicon size range of 75–150 bp. The capacity to quantify larger amplicons is applicable to the development of quantitative DNA sequencing of complex communities that often necessitate longer amplicon lengths to capture the full genetic diversity.

## Supplementary Information

Below is the link to the electronic supplementary material.

Supplementary Material 1 (DOCX 561 KB)

## Data Availability

All raw data used for final data analysis and figure generation have been uploaded to a Zenodo repository (https://10.5281/zenodo.17209789).
