# Supplementary material for: Amplification Efficiency of Quantitative PCR Reactions is Improved by Addition of Non-Target DNA
Source: Microb Ecol. 2026 Mar 17;89:84. doi: 10.1007/s00248-026-02719-0 (PMC13038649; doi:10.1007/s00248-026-02719-0)
Supplement: Supplementary file 1 [file 248_2026_2719_MOESM1_ESM.docx]

# **Supplemental Table 1.** Primers used to generate plasmid template for various amplicon sizes.

| **pCR4-amplicon size and gene target^†^** | **Primer** | **Sequence (5′–3′)** | **Reference** |
| --- | --- | --- | --- |
| 239 bp (Bacterial nitrite reductase, *nirK)* | | | |
|  | nirK876 | ATYGGCGGVCAYGGCGA | [1, 2] |
|  | nirK1040 | GCCTCGATCAGRTTRTGGTT |  |
| 265 bp (Archaeal 16S rRNA) | | | |
|  | Arch 931F | *AGGAAT TGG CGG GGG AGC A* | [3] |
|  | Arch m1100r | *BGG GTC TCG CTC GTT RCC* | [4] |
| 322 bp (Fungal nitrite reductase, *nirK*) | | | |
|  | F-nirK715F | CCDCACAACATMGACTGYCA | This study |
|  | F-nirK931R | AACTCGCTYTGCATHACGTAGTATTC | This study |
| 425 bp (Bacterial nitrogenase, *nifH*) | | | |
|  | PolF | TGCGAYCCSAARGCBGACTC | [5] |
|  | PolR | ATSGCCATCATYTCRCCGGA | [5] |
| 491 bp (bacterial nitrite reductase) | | | |
|  | nirSCd3aF | GTSAACGTSAAGGARACSGG | [6] |
|  | nirSR3cd | GASTTCGGRTGSGTCTTGA | [6] |
| 568 bp (Bacterial ammonia monooxygenase, *amoA*) | | | |
|  | amoA-1F | GGGGTTTCTACTGGTGGT | [7] |
|  | amoA-2R | CCCCTCKGSAAAGCCTTCTTC | [7] |
| 723 bp (Archaeal ammonia monooxygenase, *amoA*) | | | |
|  | Arch-amoAF | STAATGGTCTGGCTTAGACG | [8] |
|  | Arch-amoAR | GCGGCCATCCATCTGTATGT | [8] |

^†^Amplification performed with plasmid-specific primers pCR4-265F (5′–AGT CCT GCA GGT TTA AAC GAA–3′) and pCR4-312R (5’–ATA GGG CGA ATT GAA TTT AGC G–3′) that amplify the gene insert and a 69 bp portion of the plasmid.

**References**

1.	Henry S, et al. (2004) Quantification of denitrifying bacteria in soils by nirK gene targeted real-time PCR. J Microbiol Methods 59: 327-335

2.	Henry S, et al. (2005) Corrigendum to "Quantification of denitrifying bacteria in soils by nirK gene targeted real-time PCR" [J. Microbiol. Methods 59 (2004) 327-335]. J Microbiol Methods 61: 289-290

3.	Amann RI, et al. (1995) Phylogenetic identification and in situ detection of individual microbial cells without cultivation. Microbiol Rev 59: 143-169

4.	Einen J, et al. (2008) Enumeration of Archaea and Bacteria in seafloor basalt using real-time quantitative PCR and fluorescence microscopy. FEMS Microbiol Lett 282: 182-187

5.	Poly F, et al. (2001) Improvement in the RFLP procedure for studying the diversity of *nifH* genes in communities of nitrogen fixers in soil. Res Microbiol 152: 95-103

6.	Throbäck IN, et al. (2004) Reassessing PCR primers targeting nirS, nirK and nosZ genes for community surveys of denitrifying bacteria with DGGE. FEMS Microbiol Ecol 49: 401-417

7.	Rotthauwe JH, et al. (1997) The ammonia monooxygenase structural gene *amoA* as a functional marker: Molecular fine-scale analysis of natural ammonia-oxidizing populations. Appl Environ Microbiol 63: 4704-4712

8.	Francis CA, et al. (2005) Ubiquity and diversity of ammonia-oxidizing archaea in water columns and sediments of the ocean. Proc Natl Acad Sci USA 102: 14683-14688

**Supplemental Figure 1.**

**Supplemental Figure 1.** Melt curve analysis of quantitative PCR reactions targeting the bacterial ammonia monooxygenase (*amoA*) gene from circular pCR4 plasmid, soil, and genomic DNA templates. Amplification was performed on the StepOnePlus platform with PowerSYBR Master Mix without (No additive, 0 ng μL^-1^) or with pUC19 additive (Additive, 0.05 ng μL^-1^). Lines show data from individual wells for triplicate reactions with circular plasmid standards encoding the *amoA* gene (Plasmid Standards, 1×10^5^ – 1×10^1^ copies per μL^-1^ reaction), genomic DNA from *Nitrosomonas europaea* ATCC 19718 (gDNA, 2.4×10^2^ and 2.4×10^4^ *amoA* copies μL^-1^ reaction), and soil DNA from two locations. The no template qPCR controls (NTC) are shown with dashed lines. Reactions with pUC19 additive are shown in blue and additive-free in green.

# **Supplemental Figure 2.**

**Supplemental Figure 2.** Gel image of quantitative PCR reactions targeting uncut circular plasmid template with inserts of varying size. Reactions were performed on the StepOnePlus platform with PowerSYBR Master Mix containing 0 (no additive) or 2 ng μL^-1^ pUC19 additive. Gel electrophoresis was performed with 1.8% agarose, 6 μL PCR product, and 0.5 μg 100 bp ladder (New England Biolabs). Additive concentration (0, 2 ng μL^-1^) and amplicon size are shown above the wells. NTC is the qPCR no template control.
